# Multiplex Detection of B-Type Natriuretic Peptide, Cardiac Troponin I and C-Reactive Protein with Photonic Suspension Array

**DOI:** 10.1371/journal.pone.0041448

**Published:** 2012-07-27

**Authors:** Wenbin Lu, Cong Fu, Yong Chen, Jun Lu, Yuyu Yao, Chengxing Shen, Zhongze Gu

**Affiliations:** 1 Department of Cardiology, Xinhua Hospital affiliated to Shanghai Jiao tong University, Shanghai, China; 2 Department of Cardiology, ZhongDa hospital affiliated to Southeast University, Nanjing, Jiangsu, China; 3 State Key Laboratory of Bioelectronics, School of Biological Science and Medical Engineering, Southeast University, Nanjing, Jiangsu, China; University of Otago, New Zealand

## Abstract

A novel photonic suspension array has been developed for multiplex immunoassay. The carriers of this array were silica colloidal crystal beads (SCCBs). The codes of these carriers have characteristic reflection peaks originating from their structural periodicity; therefore they do not suffer from fading, bleaching, quenching or chemical instability. In addition, the fluorescence background of SCCBs is negligible because no fluorescence materials or dyes are involved. With a sandwich method, the proposed suspension array was used for simultaneous multiplex detection of heart failure (HF) and coronary heart disease (CAD) biomarkers in one test tube. The results showed that the three biomarkers: cardiac troponin I (cTnI), C-reactive protein (CRP) and B-type natriuretic peptide (BNP) could be assayed in the ranges of 0.1–500 ng/ml, 1–500 mg/L and 0.02–50 ng/ml with detection limits of 0.01 ng/ml, 0.36 mg/L and 0.004 ng/ml at 3σ, respectively. There were no significant differences between the photonic suspension array and traditional parallel single-analyte test. This novel method demonstrated acceptable accuracy, high detection sensitivity and reproducibility and excellent storage stability. This technique provides a new strategy for low cost, automated, and simultaneous multiplex immunoassays of bio-markers.

## Introduction

Acute myocardial infarction (AMI) is a major and growing public health problem that frequently leads to irreversible heart failure (HF) and is a leading cause of death each year [1]. Despite the high mortality rate, many of these deaths can be avoided by early detection and intervention. C-reactive protein (CRP) [2], [3], B-type natriuretic peptide (BNP) [4] and cardiac troponin I (cTnI) [5], [6] can respectively serve as important markers of plaque stability, HF, and myocardial injury, but also collectively serve as prognostic indicators of outcome after AMI. However, the use of any single biomarker is not sufficient to accurately assess coronary heart disease (CAD) and HF. Thus, multiplex immunoassay of biomarkers has attracted considerable interest to meet the growing demand for diagnostic applications in the process of CAD [7]. Multiplex immunoassays are also advantageous because the offer higher sample throughput, less sample consumption, reduced turnaround times and a more reasonable cost compared to the traditional parallel single-analyte immunoassay [8].

Multiplex immunoassay, which is based on molecules binding or recognition to distinguish different binding events, has been used for the detection and quantification of a broad variety of analyses in clinical diagnosis [9], [10]. Planar array is the most common and versatile encoding method [11], [12], in which the probe molecules are immobilized on a substrate and encoded by coordinate of their position [13]. A major drawback of this planar array is the long assay time that is due to the diffusion-limitation of the molecular binding kinetics on the substrate. In recent years, suspension arrays have demonstrated high flexibility, fast reaction and good repeatability [14], [15]. These arrays also consume less analyte sample and subsequently can be performed at a lower cost [13]. Among the various suspension arrays available for use, spectrum-encoded micro-particles are often utilized because of their simplicity in both encoding and detection [16], [17], [18]. Fluorescent dyes [19], [20] and quantum dots [21], [22] are the main spectrum-encoding elements, however these present with several limitations; the fluorescence dyes tend to be quenched and the quantum dots are often bio-toxic [23], [24].

For these reasons, we proposed silica colloidal crystal beads (SCCBs) as encoded supports for suspension array to detect three biomarkers. SCCBs are self-encoded through the characteristic reflection peak originated from its stop-band of colloid crystal [25]. The code is very stable due to the peak position based on its periodical structure. In addition, higher surface-to-volume ratios lead to more fluorescence dyes that participate in the immunoassay. Collectively, these properties make the photonic suspension array suitable for high sensitive and high throughput detection [8]. In this paper, we proposed to use a suspension array for the multiple detection of three heart injury markers: cTnI, CRP and BNP.

## Materials and Methods

### Materials

Human cTnI, CRP were purchased from Biovision, California, USA. BNP was obtained from GenScript, New Jersey, USA. Mouse monoclonal anti-human cTnI antibody, anti-human CRP antibody, anti-human BNP antibody and fluorescent isothiocyanate (FITC) tagged goat anti-human cTnI, anti-human CRP and anti-human BNP were obtained from Gene Tex Co., Southern California, USA. Bovine serum albumin (BSA) was purchased from Sigma Chemicals, Perth, Australia. 3-glycidoxypropyltrimethoxysilane (GPTMS) and Toluene were received from Alfa Aesar Co. Lancashire, UK. Monodisperse silica Nan particles were synthesized by the StÖber method [26]. Clinical serum samples were collected from patients who suffered from stable angina pectoris, unstable angina pectoris confirmed by coronary arteriography, or remote myocardial infarction confirmed by electrocardiogram (ECG). Patients were excluded if they had severe hepatic or renal insufficiency. All samples were obtained from patients in ZhongDa Hospital affiliated with Southeast University, China. The studies were approved by ethics committee of ZhongDa hospital affiliated to Southeast University, Nanjing, Jiangsu, China and performed according to the Declaration of Helsinki and obtained written consent from all participants involved in our study.

### Instrumentation

The microstructures of the SCCBs were characterized by a scanning electron microscopy (SEM, Hitachi, S-300N). Photographs of the SCCBs were taken with an optical microscope (Olympus BX51) equipped with a CCD camera (Media Cybernetics Evolution MP 5.0). Reflection spectra of the SCCBs were recorded by a microscope equipped with a fiber optic spectrometer (Ocean Optics, USB2000). Fluorescence spectra of SCCBs were recorded by a microscope equipped with a fiber optic spectrometer (Ocean Optics, QE65000).

### SCCBs generation

The micro-fluidic device used for SCCB generation was custom made. The concrete procedures were described in previous study [27]. The SCCBs were fabricated by micro-fluidic device which was composed of syringe pump, PTFE pipe and needle. This device was easily operated and can produce stable size-controlled SCCBs. A series of beads with different reflection peaks was obtained by modulating the microstructure. To fulfill the demand of multiplex immunoassay, three kinds of aqueous suspension containing mono-disperse silica Nan particles with diameters of 290 nm, 314 nm and 375 nm were used for the colloidal crystal beads fabrication. Subsequently, three kinds of 180 um SCCBs with the reflection peak position at 465, 502 nm and 600 nm were made.

### Functionalized of suspension array

**Figure 1:**
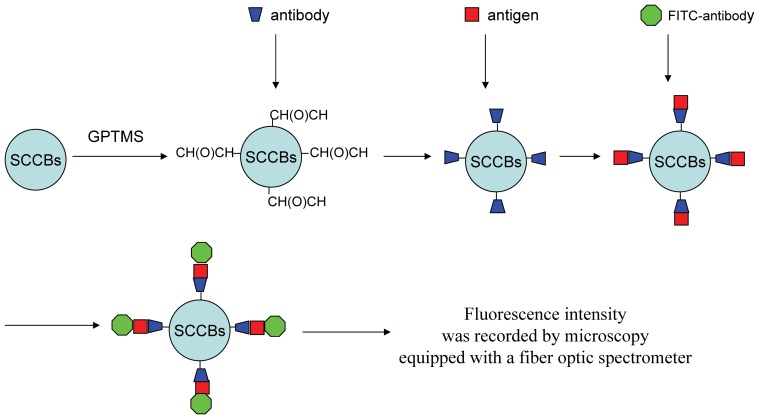
Scheme of the brief mechanism of how the silica colloidal crystal beads were functionalized. The surface of SCCBs can be chemically modified by 3-glycidoxypropyltrimethoxysilane(GPTMS), thus protein amino can bond to surface of SCCBs. More analyte bond to specific antibody fixed on the surface of SCCBs can combine to more fluorescence-conjuncted reagent, more fluorescence signal can be captured by proper equipment.

A brief mechanism of how the suspension array works is shown in **figure 1**. We introduce GPTMS to fulfill the modification of the SCCBs surface [28]. GPTMS is a frequently used silicon-alkane couple reagent. It has methoxy which can bond to inorganic material (e.g. silicon) and epoxy group [-CH(O)CH-] which can bond to organic material. The surface of SCCBs can be chemically modified by GPTMS, thus protein amino can bond to surface of SCCBs due to the epoxy group after modified by GPTMS. Consequently, a quantitative sandwich method possibly be established with SCCBs carrier. Generally, more analyte bond to specific antibody fixed on the surface of SCCBs can connect to more fluorescence-conjuncted reagent, more fluorescence signal can be captured by proper equipment.

### Probes immobilization

Antibody probes (anti-human cTnI antibody, anti-human CRP antibody, anti-human BNP antibody) were immobilized on SCCBs through the covalent bonding method. Firstly, the SCCBs were treated with the solution (30% hydrogen peroxide and 70% concentrated sulfuric acid) for 48 h. After this the SCCBs were washed with distilled water and dried by nitrogen flow, and then subsequently treated with a toluene solution of GPTMS (10%) for 24 h. Following this, the SCCBs were incubated with antibody probes (0.1 mg/mL for anti-human cTnI and anti-human-BNP, 1 ug/mL for anti-human CRP) in PBS buffer at 4°C for 12 h. SCCBs were then washed with toluene, dehydrated alcohol and distilled water in turn. Finally, the un-reacted epoxy groups on the SCCBs' surface were passaged with 5% BSA PBS buffer for 2 h at 37°C. For multiplexed immunoassays, three kinds of these SCCBs, with the reflection peak position in 465, 502 and 600 nm, were modified with mouse monoclonal anti-human cTnI antibody, anti-human CRP antibody, anti-human BNP antibody, respectively.

### Detection of bio-markers

For single analysis, different concentrations of human proteantigen were used to incubate with antibody modified on SCCBs (2 ul per bead) in the test tubes for 30 min at 37°C and unconjugated proteantigen was washed away by PBS buffer. Then, FITC-goat anti-human antibody (0.1 mg/mL for FITC-goat anti-human cTnI and FITC-goat anti-human-BNP, 1 ug/mL for FITC-goat anti-human CRP) was administered to the test tubes and incubated for another 30 min while being shaken at 37°C. Fluorescence spectra of SCCBs were measured after thoroughly washing with PBS buffer. Replication at any concentration was 5. The detection limit was calculated from the zero calibrator plus three times of standard deviation. For multiplexed detection of bio-markers, three kinds of SCCBs, with the reflection peak position at 465, 502 and 600 nm, immobilized with anti-human TnI antibody, anti-human CRP antibody, anti-human BNP antibody were put into one test tube. Then the serum sample suspension was added. After being shaken for 30 min at 37°C and washed with PBS buffer, the SCCBs were incubated with the mixed solution containing the three FITC-anti-human marker antibodies. In the end, the SCCBs were thoroughly washed with PBS buffer and put into the beads chamber for decoding and bio-reaction detection.

### Statistical analysis

All biomarker detections by electro chemiluminescence immunoassay (ECLIA) were carried out automatically with the Modular analytics (Roche). Correlation between ECLIA and our suspension array was calculated by Spearman regression analysis. All analyses were performed with SPSS 13.0 software.

## Results

### Design of suspension array

**Figure 2:**
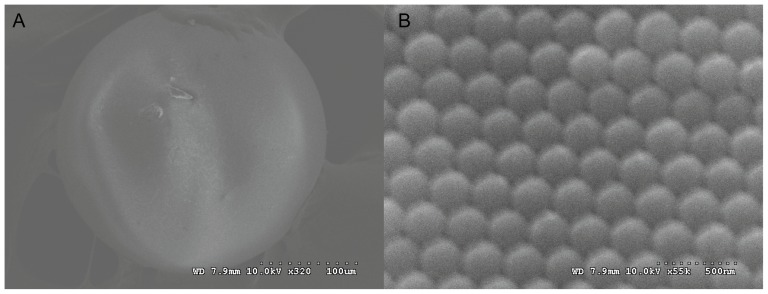
Scanning electron micrograph images of Silica colloidal crystal beads composed of 375 nm nanoparticles. A) Low magnification images of a bead with a diameter of 180 µm. B) High magnification images of the bead surface showing the hexagonal alignment of nano-pariticles.

SCCBs are derived from the assembly of mono-disperse colloidal nano-pariticles in droplet templates. As the SCCBs are derived from the assembly of mono-disperse colloidal nano-pariticles in droplet templates, The surfaces of SCCBs and ordered hexagonal symmetry of the nano-particles were shown in **figure 2A and 2B**. To examine the high-throughput application of the photonic suspension array, we used the microscope equipped with a fiber optic spectrometer for SCCB decoding and immunoreactions. When the SCCBs were exposed to the white light under normal incidence through the microscope, the reflection peaks could be detected and the wavelength of the peak could be recorded as the peak position for decoding. When the white light was replaced by blue light with the wavelength at 488 nm, the fluorescence spectra of the SCCBs were recorded for the subsequent analysis.

### Optimization of analytical conditions

**Figure 3:**
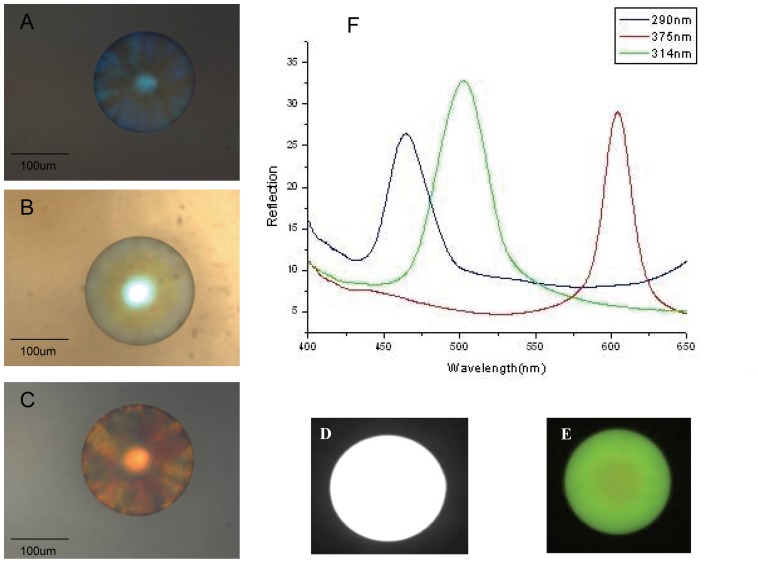
Microscope image of three SCCBs with or without FITC-antibody conjuncted and optics property. **A**) Three dimensional image of SCCBs composed of silica nanoparticles with the diameter of 290 nm. **B**) Three dimensional image of SCCBs composed of silica nanoparticles with the diameter of 314 nm. **C**) Three dimensional image of SCCBs composed of silica nanoparticles with the diameter of 375 nm. **D**) Black and white photograph of FITC-antibody conjuncted SCCBs composed of silica nanoparticles with the diameter of 290 nm. **E**) Color photograph of FITC-antibody conjuncted SCCBs composed of silica nanoparticles with the diameter of 290 nm. **F**) Reflection spectra of the three kinds of SCCBs.

To maintain the high sensitivity and improve the accuracy, three kinds of the uniform 180 um multicolor SCCBs (**Fig. 3**), with their reflection peak position in 465,502 and 600 nm were used for the detection of bio-markers after AMI in the experiments. Black and white as well as color images of FITC-antibody conjugated SCCBs were captured by the optical microscope and CCD camera. Reflection peaks of three SCCBs were observed by microscope equipped with a fiber optic spectrometer.

**Figure 4:**
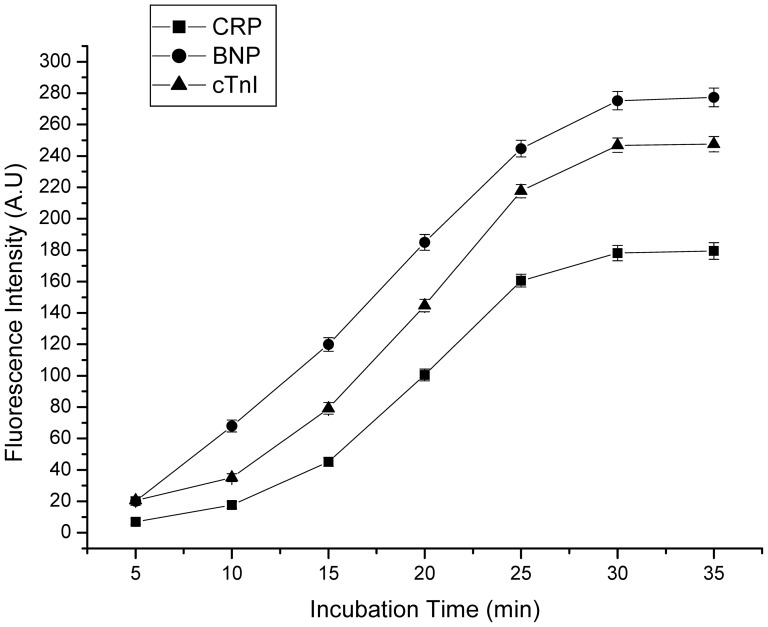
Effects of incubation time on fluorescence intensities for 100 mg/L CRP, 10 ng/mLBNP, 100 ng/mL cTnI. The number of repeated experiments at any concentration was five. Error bars represent standard deviations.

We tested the relationship between the fluorescence intensities and incubation times of three markers. With an increasing incubation time, all the fluorescence intensities for cTnI (100 ng/ml), CRP (100 mg/L), and BNP (10 ng/ml) quickly increased and reached their maximum values at 30 min (**Fig. 4**). At the incubation time of 30 min, the fluorescence signals were stable at the maximum values for each proteantigen.

### Evaluation of cross-reactivity

**Figure 5:**
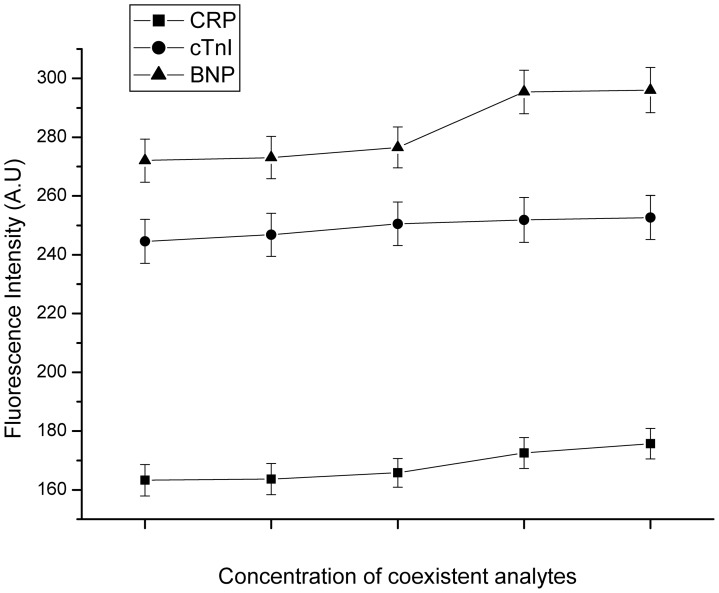
The cross-reactivity among the three markers and their non-cognate antibodies. They were examined by comparing the fluorescence signals at a definite concentration of CRP (100 mg/L), cTnI (100 ng/mL) and BNP (10 ng/mL) with increasing levels of other two analytes, The five scale points represent the five concentrations of coexistent analytes for each protein respectively. Cross-reactivity among CRP specific antibody and BNP, cTnI was measured after 100 mg/L CRP incubated with specific CRP antibody and different concentrations of BNP and cTnI(with concentration gradient 10, 20, 30, 40, 50 ng/mL). Cross-reactivity among cTnI specific antibody and CRP, BNP was measured after 100 ng/mL cTnI incubated with specific cTnI antibody and different concentrations of CRP and BNP (with concentration gradient 1, 2, 3, 4, 5 mg/L). Cross-reactivity among BNP specific antibody and CRP, cTnI was measured after 10 ng/mL BNP incubated with specific BNP antibody and different concentrations of CRP and cTnI (with concentration gradient 1, 2, 3, 4, 5 mg/L). The number of repeated experiments was five. Error bars represent standard deviations.

We showed the effects of the coexistent analyses on the fluorescence signal for each marker at a concentration of 100 ng/mL for cTnI, 100 mg/L for CRP and 10 ng/ml for BNP (**Fig. 5**). Even when the concentrations of the interferents reached 50 ng/ml or 5 mg/L for each, the maximum changes of the fluorescence signals for cTnI (100 ng/ml), CRP (100 mg/L), and BNP (10 ng/ml) did not go beyond 3.3%, 7.6% and 8.8%, respectively.

### Experimental performance of the photonic suspension array for bio-markers

**Figure 6:**
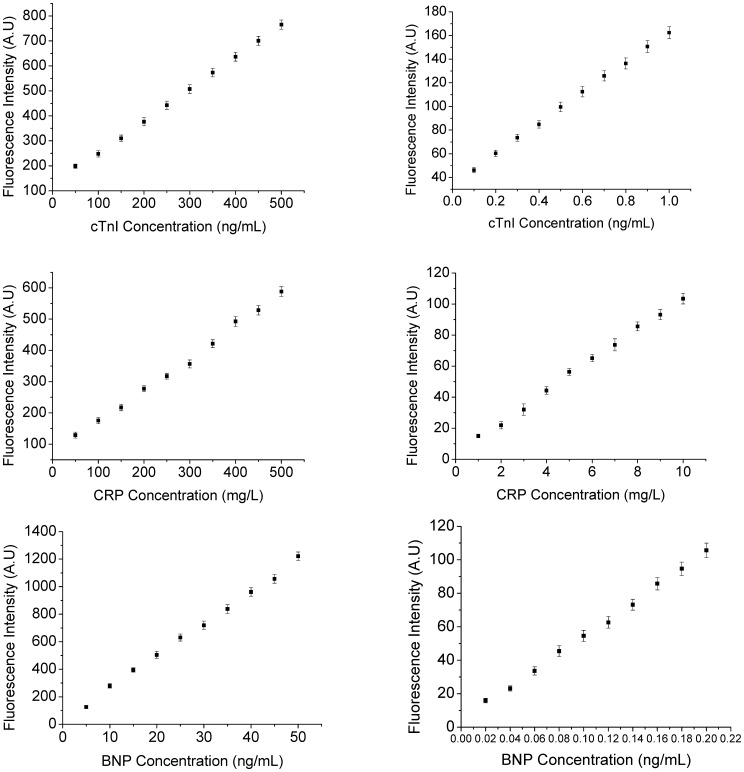
Calibration plots of fluorescence intensity vs. biomarkers concentrations. **A**) High (left) and low (right) concentration of cTnI curves. **B**) High (left) and low (right) concentration of CRP curves. **C**) High (left) and low (right) concentration of BNP curves. The number of repeated experiments at any concentration was five. Error bars represent standard deviations.

We assayed routine samples of different concentrations of three markers from 0 to 100 ng/ml or mg/ml under optimal conditions. The dose-response and calibration curves for the multiplex immunoassay of cTnI, CRP, and BNP are shown in **Figure 6**. The curves were consistent with those commonly observed for immunoassays and we used curve-fitting for the calibration procedure. The limits of detection at a signal-to-noise ratio of the three bio-markers were 0.01 ng/mL, 0.36 mg/L and 0.004 ng/mL.

To investigate the reproducibility of the newly prepared photonic suspension array, we repeated the assays five times for two different concentrations of three bio-markers, 5 mg/L or 100 mg/L for CRP, 0.1 ng/mL or 10 ng/mL for BNP and 0.5 ng/mL or 100 ng/mL for cTnI. The coefficients of variation (CVs) among the five repetitions were 2.5% for 5 mg/L CRP and 1.5% for 100 mg/L CRP; 2.8% for 0.1 ng/mL and 0.4% for 10 ng/ml BNP; 1.2% for 0.5 ng/mL and 0.8% for 100 ng/ml cTnI. For conventional method, the CVs among the five repetitions were 4.2% for 5 mg/L CRP and 3.5% for 100 mg/L CRP; 5.1% for 0.1 ng/mL and 2.3% for 10 ng/ml BNP; 3.7% for 0.5 ng/mL and 3.1% for 100 ng/ml cTnI. We also tested the non-specific absorption fluorescence intensity, and found that the non-specific was less than three times the background. When the photonic suspension array was not in use, the SCCBs were stored in PBS (pH 7.4) at 4°C. There were no obvious changes after storage for at least one year for the SCCBs without probe immobilization and at least one week for the SCCBs with probe immobilization.

### Clinical application of photonic suspension array

**Figure 7:**
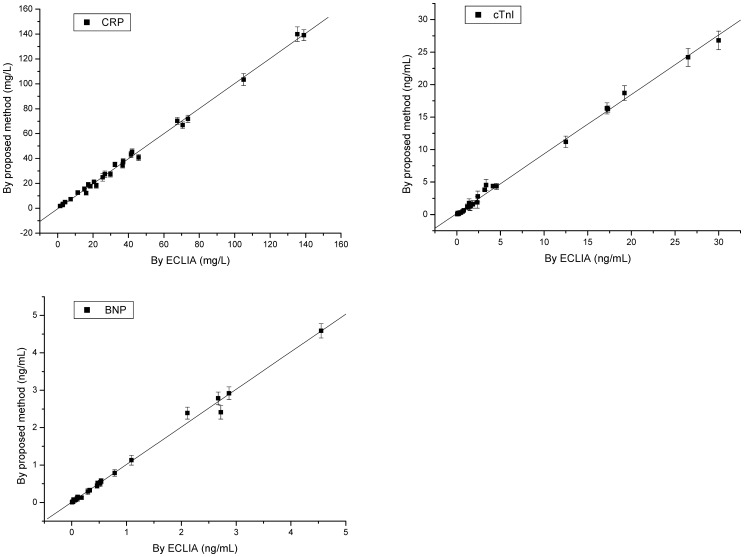
Correlation between the photonic suspension array and the standard ECLIA method for the three bio-marker measurements of 27 clinical samples. The number of repeated experiments of every clinical sample for any bio-marker was five. Error bars represent standard deviations.

To investigate the analytical reliability and application potential of the photonic suspension array for a multiplex immunoassay in clinical analysis, this array was compared with the commercially proven ECLIA method; the latter was carried out with parallel single analysis as a reference. We examined 27 clinical serum samples. Whole blood samples were collected using the standard vein-puncture technique followed with centrifugation of 1000 rpm for 15 minutes. The results from this analysis are shown in **Figure 7**. The regression equations (linear) for these data are as follows (x axis, ECLIA; y axis, photonic suspension array).


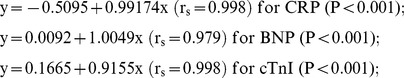


## Discussion

Hypersensitivity and high-throughput applications are the two most important advantages of suspension arrays. Generally, the sensitivity and accuracy of the suspension array can be affected by the character of the probe carriers, antibodies and detectors. For our suspension array, the particular hexagonal structure of SCCBs carriers is the most novel property and this may have beneficial effects on the sensitivity of the test. This hexagonal surface structure permits more molecules to be immobilized on beads and participated in immunoreactions. This structure also provides a nano-patterned platform that can reduce the steric hindrance of molecules. Therefore, a greater number of molecules on this platform are free to react with their specific complements, which substantially increases the efficiency of the immunoreactions.

In the process of decoding and bio-reaction detection, the size of the SCCBs had the greatest effect on the suspension system. Sizes that were too large or too small both influenced the accuracy and efficiency of immuno-detection through suspension array. Furthermore, the incubation time had a detectable effect on the process of immunoreactions. Incubation times of the immunoreactions that were excessive or too short enlarged variance in or between analytical batches or resulted in failure of the experiment. The successful development of the multiplex immunoassay required that the common incubation time must be suitable for all analyses. Our results showed that the size of the SCCBs fit for the suspension array detection. We also have found the optimal reaction time indicating the maximum formation of these sandwich immunocomplexes. The optimal incubation times of our photonic suspension array for forming these sandwich immunocomplexes were much shorter than those of 1–3 h at 37°C for the conventional micro well plates of ELISA method. Generally, incubation time depends on the kinetic characteristics of immunoreactions and mass transfer of immunoreagents. The use of photonic suspension arrays with consecutive shake at 37°C not only reduced the steric hindrance of the bio-molecules, but also increased the reaction kinetics. This protocol produced a short diffusion distance for the immunoreagents and thus accelerated their mass transport and increased the immunoreactions' rate.

Cross-reactivity is a crucial analytical parameter related to the specificity and reliability of the multiplex immunoassay. In our suspension array, three markers were detected in one test tube and the cross reactivity that potentially occurred among the markers and their non-cognate antibodies were important factors influencing the reliability of the proposed system. Here, the cross-reactivity among the three proteantigen and their non-cognate antibodies was examined by comparing the fluorescence signals at a definite concentration of specific analyses with increasing levels of the other two coexistent analyse. The results of three markers cross-reactivity indicated that the cross-reactivity between antibodies and their non-cognate antigens was negligible and the three markers could be detected in one test tube without noticeable interference.

Sensitivity and detection ranges are both crucial for biomarkers detection. Low limits of detection enhance the sensitivity of immunoassay. The limits of detection of our array were low compared with traditional method (0.742 mg/L for CRP, 0.05 ng/mL for cTnI and 0.005 ng/mL for BNP). Therefore, the sensitivity and detection ranges of our suspension array were sufficient for practical applications.

Reproducibility of the newly prepared photonic suspension array was also measured in our experiment. The data demonstrate acceptable detection and fabrication reproducibility of the proposed immunoassay system. non-specific absorption fluorescence intensity was also negligible. These results suggest that the realization of photonic suspension array depended multiplex immunoassay of biomarkers.

The comparison of photonic suspension array and commercially proven ECLIA method showed no significant difference between the results of the two methods, demonstrating the good analytical reliability of our method. In addition, the photonic suspension array consumed less analyte sample than the ECLIA, and fulfilled the multiplex detection of the three markers in one test tube, which simplified the immunoassay process.

## Conclusion

A novel multiplex immunoassay based on silica colloidal crystal beads was established for the detection of biomarkers of plaque stability, HF and myocardium injury after AMI. The suspension array showed high sensitive, good detection reproducibility and storage stability. Our suspension array using SCCBs as coding elements was stable and avoided quenching and bleaching due the fact that no fluorescence dyes were involved. The surface structure of SCCBs overcomes the steric hindrance of the bio-molecules and permits a simple, stable, high throughput and sensitivity assay. Our data show that the suspension array is suitable for multiplex detection of biomarkers of plaque stability, HF and myocardium injury after AMI and the values obtained from our method were consistent with the established ECLIA method. This article presents a novel method of multiplex immunoassay that can be further developed for application in disease diagnosis.
